# Towards controlling the crystallisation behaviour of fenofibrate melt: triggers of crystallisation and polymorphic transformation†

**DOI:** 10.1039/c8ra01182f

**Published:** 2018-04-10

**Authors:** Pratchaya Tipduangta, Khaled Takieddin, László Fábián, Peter Belton, Sheng Qi

**Affiliations:** School of Pharmacy, University of East Anglia Norwich Norfolk NR4 7TJ UK sheng.qi@uea.ac.uk; School of Chemistry, University of East Anglia Norwich Norfolk NR4 7TJ UK; Department of Pharmaceutical Sciences, Faculty of Pharmacy, Chiang Mai University Chiang Mai Thailand 50200

## Abstract

Fenofibrate (FEN) is a dyslipidemia treatment agent which is poorly soluble in water. FEN has tendency to form polymorphs and its crystallisation behaviour is difficult to predict. The nucleation process can be initiated by mechanical disruption such as ball milling or surface scratching which may result in different crystallisation behaviour to that observed in the unperturbed system. This study has obtained insights into the controllability of FEN crystallisation by means of regulating the exposed surface and growth temperatures during its crystallisation. The availability of an open top surface (OTS) during the crystallisation of the FEN melt resulted in a mixture containing FEN form I and IIa (I ≫ IIa) at room temperature, and in the range 40 to 70 °C. Covering the surface led to significant increases in the yield of form IIa at room temperature and at 40 and 50 °C. These temperatures also yielded the highest amount of form IIa in the OTS samples whilst crystallisation at 70 °C led to only FEN form I crystals regardless of the availability of the free surface. The metastable FEN form IIa transforms to the stable form I under the influence of a mechanical stress. Additionally, the introduction of OTS before the completion of crystallisation of form IIa led to a ‘switch’ of from IIa growth to form I. This study demonstrates that the polymorph selection of FEN can be obtained by the manipulation of the crystallisation conditions.

## Abstract 2

Fenofibrate is a dyslipidemia treatment agent. Its crystallisation behaviour is difficult to predict. This study investigated the controllability of its crystallisation by means of regulating the exposed surface and growth temperatures.

## Graphical abstract

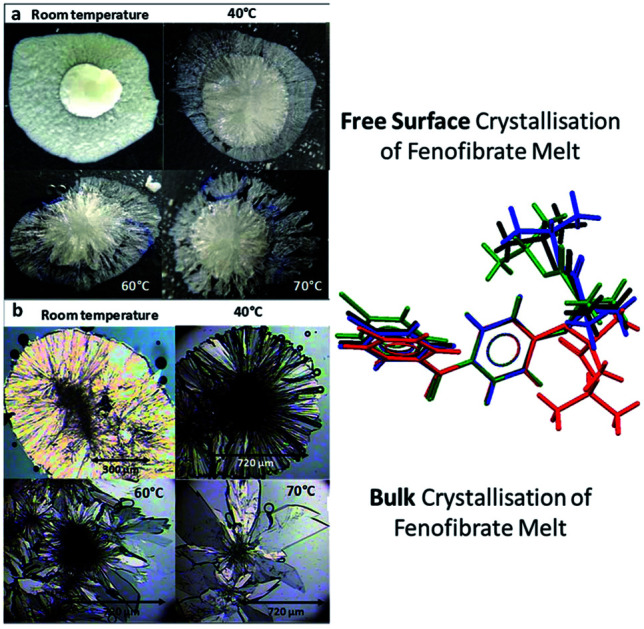

## Introduction

Fenofibrate (propan-2-yl 2-(4-[(4-chlorophenyl)carbonyl]phenoxy)-2-methylpropanoate) has been prescribed to treat hypercholesterolemia since 1970.^1^ It is a prodrug in which fenofibric acid is the active form. The pharmacological effect of fenofibrate (FEN) is to reduce low-density lipoprotein (LDL) and increase high-density lipoprotein (HDL) by binding to peroxisome proliferator-activated receptor alpha (PPARα).^1,2^ FEN is categorised as a BCS class II drug because of its poor aqueous solubility (0.8 μg ml^−1^), but it has a high permeability (>90%) through lipid membranes.^3,4^ To overcome the solubility issue, particle size reduction (micronized and nanocrystals) and lipid dispersions have been used in the commercialised formulations containing FEN.^1^ With the increased use of the amorphous state of the drug in dispersion based formulations,^5–8^ it is extremely important to fully understand the stability and crystallisation behaviour of amorphous FEN. Zhou and co-workers have conducted a systematic study of a range of low-molecular weight drugs including FEN^9^ probing the relationship between physical stability and a range of thermodynamic properties. They identified two important properties: molecular mobility, which is a measure of the rate of cooperatively rearrangement in subsystems in the amorphous state, and configurational entropy, which is the entropy difference between the disordered state and the crystalline state. The study revealed that the molecules that have high configurational entropies and low molecular mobilities were the least likely to spontaneously crystallise.^9^ In amorphous FEN the configurational entropy had a relatively high value of 76.6 J mol^−1^ K^−1^ (relative to form I), but it also exhibited a relatively high molecular mobility value of 72.3 × 10^−5^ s^−1^.^9^ The high molecular mobility can possibly be attributed to weak intermolecular interactions,^10,11^ which results in the low probability of FEN molecules forming stable nuclei and heterogeneous nucleation is the main route to initiate the crystallisation.^12^ Heterogeneous nucleation triggers, including scratching the surface or placing an FEN seed on the surface of amorphous FEN, resulted in an immediate initiation of crystallisation.^12^

Three FEN polymorphs have been documented in the Cambridge Structural Database (CSD). The stable form I (with a melting point at 80 °C) is the standard polymorph used as API in commercial tablets and capsules. Form I can be obtained by a slow solvent evaporation method.^13^ The metastable form called form II was reported by Di Martino *et al.* and Heinz *et al.*^14–16^ It was produced by the recrystallization of amorphous FEN from the melt. The melting point of the product was 74 °C. It converts to the stable form I during heating *via* melt recrystallization and melts again at 80 °C.^14–16^ Balendiran and co-workers reported the single crystal structure of a FEN form they called form II. It was recrystallised by a solvent (ethanol) evaporation method, but no melting point and spectroscopic data were reported.^17^ However, it is not clear that the two types of form II are the same. In order to avoid confusion, the FEN form II from Di Martino *et al.* and Heinz *et al.* will be addressed as FEN form IIa in this study and the one from Balendiran will be addressed as form IIb. Form III was first reported by additive induced crystallisation using talc and it is not possible to crystallise it in isolation as pure form.^11^ The characteristic data of all the metastable FEN forms available from the literature: IIa, IIb and III are summarised in Table 1.

**Table 1:** Summary of the available literature data related to metastable FEN form II and III

Characteristic data of FEN form II	Assignment by the authors	References	Characteristic data of FEN form III	Assignment by the authors	Reference
DSC (*T*_m_ at 74 °C)	IIa	14,16	DSC (*T*_m_ at 50 °C)	III	11
PXRD pattern	IIa	14	PXRD pattern	III	11
Raman spectrum	IIa	16	FTIR spectrum	III	11
Single crystal	IIb	17	Single crystal	III	11

This study aims to investigate the effect of the availability of an open top surface (OTS) and variations in incubation temperature on the crystallisation behaviour of FEN melt. There is good evidence that allowing crystallisation to proceed from the melt in an unconstrained manner (the so called free surface) can increase the rates of crystal growth in drugs such as nifedipine, indomethacin, and griseofulvin.^18–21^ This increased growth rate is thought to be due to higher molecular mobility at free surfaces.^18–21^ Increasing numbers of pharmaceutically related molecules have been reported to exhibit surface-induced polymorph selection behaviour when crystallised from solvents and subjected to different solid substrate surfaces.^22–25^ However, little has been reported in terms of whether the free surface has an impact on polymorph selection of the crystals obtained from melt-cooled amorphous materials. Furthermore, the temperature during crystallisation is another important factor that influences the formation of crystal polymorphs.^26^ Therefore this study further investigated the combined effect of growth temperature and availability of free surface on the crystallisation behaviour of amorphous FEN. As the homogeneous nucleation of amorphous FEN is a slow process,^12,27^ the heterogeneous nucleation and crystallisation of FEN was initiated by scratching the surface of amorphous FEN using a metal spatula.

## Materials and methods

### Materials

Crystalline FEN form I was kindly donated by Merck Serono, Germany. Glass slides and coverslips 24 × 24 mm, thickness 0.16–0.18 mm were purchased from Academy Science Limited (Kent, UK).

### Preparation of FEN crystallisation samples

Approximately 3–5 mg of crystalline FEN form I powder was placed on a glass slide and heated on a hot plate at 100 °C until it was completely molten. The glass slide was immediately removed from the hot plate to allow the sample to cool to room temperature. The surface disruption technique, involving the use of a stainless-steel spatula to scratch the surface of the amorphous FEN, was used to induce crystallisation. After surface scratching either of two procedures was followed. In the first, a cover slip was immediately placed on top of the sample to sandwich the drug between the glass slide and the cover slip (these samples are referred to as “bulk crystalline FEN samples” or B-FEN in this study). The second set of scratched samples were left to crystallise with an OTS (these samples are referred to as “free surface crystalline FEN samples” or FS-FEN). In these samples, where it was possible, examination was made of the top and bottom layers of the material separately. Immediately after the initiation of crystallisation by surface scratching, both B-FEN and FS-FEN samples were incubated in an oven at 40, 50, 60 or 70 °C for 30 min to obtain complete crystallisation. The storage stability studies of B-FEN and FS-FEN were conducted by storing the samples at ambient temperature in sealed glass vials for up to 6 months. For the storage stability of B-FEN samples, once the crystallisation was completed, the top cover slip was carefully removed prior to the storage.

### Differential scanning calorimetry (DSC)

A DSC Q2000 TA (New Castle, USA) which was equipped with an RSC 90 cooling unit was used. Full temperature and heat capacity calibrations were performed prior to the sample measurement. The scanning rate used was 10 °C min^−1^ over a temperature range of −60 °C to 120 °C. For isothermal modulated temperature DSC (MTDSC) at 60 and 70 °C for 120 minutes, the amplitude of ±2 °C with a period of 60 seconds was used for all measurements. The dry nitrogen was purged at 50 ml min^−1^. Between 1 and 3 mg of samples was weighed out accurately and encapsulated in TA standard crimped pans. All measurements were performed in triplicate.

### Polarised light microscopy (PLM) and hot-stage polarised light microscopy (HS-PLM)

PLM model Leica DM LS2 (Wetzlar GmbH, Germany) equipped with a JVC camera with 5, 10 and 20× magnification was used to study the morphology of the crystalline FEN. The microscope was controlled from Software Studio Capture 1.6 from Mettler Toledo Ltd (Greifensee, Switzerland). The growth rate of FEN was analysed by using ImageJ software. The average values of three measurements from different images were used. A Hot-stage HS82 with a controller by Mettler Toledo Ltd (Greifensee, Switzerland) was used to identify the polymorphic form of the B-FEN (70 °C) by a visual observation of the melting point using a heating rate of 20 °C min^−1^.

### Attenuated total reflection Fourier transform infrared spectrometry (ATR-FTIR)

Infrared spectra in this study were acquired by a Bruker IF-66 spectrometer (Bruker Optics, Coventry, UK) equipped with a golden gate MKII accessory from Specac Ltd (Orpington, UK). A few mg of the sample was placed on the crystal of the ATR cell; each spectrum was acquired at a resolution of 2 cm^−1^ with 32 scans over a range of 4000 to 550 cm^−1^ at ambient temperature. For the hot-stage ATR-FTIR, the spectra were acquired every 5 minutes for 3 hours when the sample was placed on the heated ATR stage at 70 °C. The spectra were baseline corrected by using the Bruker OPUS software (Bruker Optics, Coventry, UK). At least three ATR-FTIR spectra were acquired from different sites of each sample to ensure homogeneity throughout the sample tested.

### Powder X-ray diffraction (PXRD)

A Thermo ARL Xtra model (Ecublens, Switzerland) X-ray diffractometer was used in this study. The glass cover slips on which the FEN crystals were grown were placed into the sample holder. The crystals were not milled into powder form in order to avoid any polymorphic transformation induced by milling. However, it is worth highlighting that as the samples were not in powder form, orientation effects may affect the diffraction patterns obtained in this study. The X-ray source generated from a copper X-ray tube (1.540562 Å) set at 45 kV and 40 mA. Data were collected over a 2*θ* range of 5 to 50°, with a step size of 0.01° with 1 second per step. The instrument was operated under ambient conditions.

### Single crystal X-ray diffraction

The fully crystallised OTS-FEN sample, grown at 40 °C, was used for conducting single crystal X-ray diffraction experiments of FEN form IIa. The diffraction experiments were performed using an Oxford Diffraction Xcalibur-3/Sapphire3-CCD diffractometer (Oxford Diffraction Ltd., Oxford, UK) equipped with a graphite monochromator using Mo-Kα radiation (*λ* = 0.71073 Å). A single crystal was selected from crystals of FEN form IIa which were colourless prisms. This single crystal sample was mounted on a glass fibre under oil and fixed in the cold nitrogen stream on the diffractometer. Intensity data were measured by thin-slice ω- and φ-scans at 140 K. The diffraction data were processed using the CrysAlisPro-CCD and -RED programmes.^28^ The structure was solved in SHELXT^29^ using the dual-space approach and refined with SHELXL^30^ as implemented in ShelXle GUI.^31^ The non-hydrogen atoms were refined with anisotropic thermal parameters. Hydrogen atoms were included in idealised positions and allowed to refine isotropically.

## Results and discussion

### Verification of form IIs by single crystal structure analysis

**Fig. 1:**
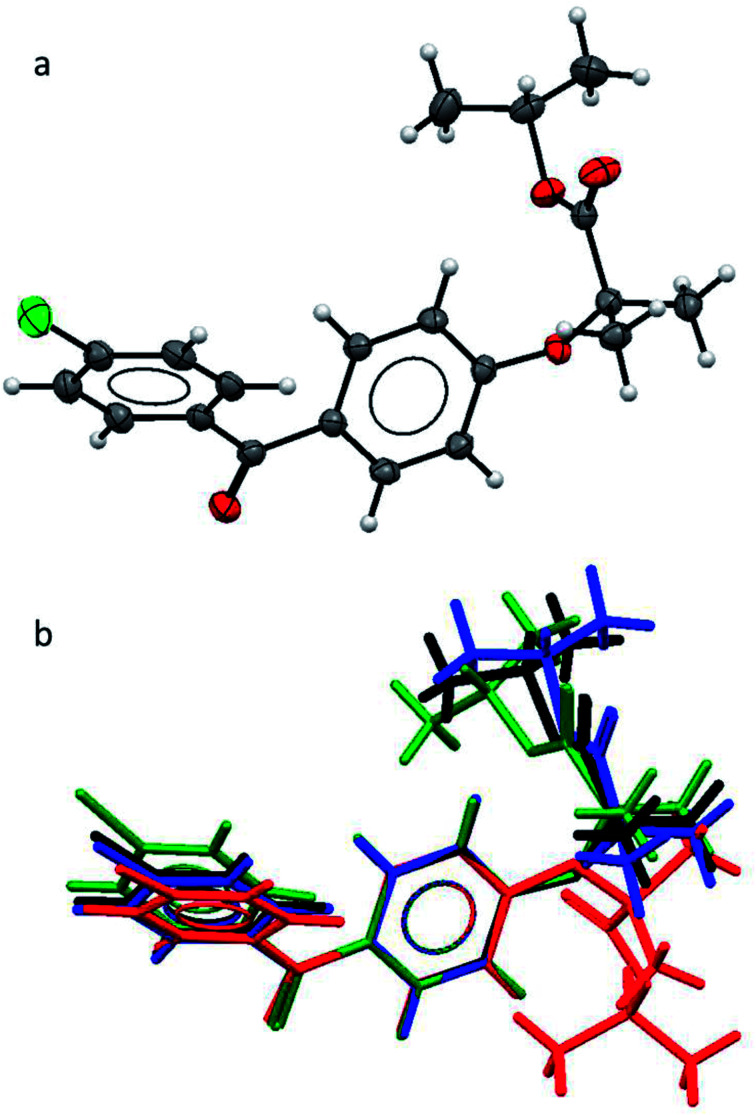
(a) ORTEP structure of form IIa; (b) an overlay of the molecular conformations in FEN form I (black), form IIa (blue), form IIb (red), form III (green).

As the polymorphic form is the focus of this study, we first needed to verify the structural difference of the two types of form II. As mentioned earlier, in the literature, form IIa was reported to be obtained by melt-crystallisation.^14–16^ Therefore form IIa was prepared by melt-crystallisation and the melting point and FTIR spectra were used to verify the polymorph being the same as the one reported in the literature. The single crystal structure of form IIa was also determined. The crystallographic parameters of form IIa in comparison with the other known forms are shown in Table 2. The FEN molecule can be divided into two main parts: two aromatic rings linked by a keto group and flexible aliphatic tail. An ORTEP drawing of the molecular structure of FEN form IIa is shown in Fig. 1a. To compare form IIa to the other reported forms (I, III and IIb), their molecular structures were overlaid as illustrated in Fig. 1b. The conformation of FEN in form IIa is largely similar to forms I and III, while the orientation of the alkyl fragment relative to the aromatic rings is markedly different in form IIb. The angles between the planes of the two aromatic rings in forms I, III and IIa are 48.62(7)°, 45.73(9)° and 48.25(10)°, respectively; whereas it is 53.73° in form IIb. This further confirms that the two form IIs reported in literature are structurally different.

**Table 2:** Crystallographic parameters of known polymorphic forms of FEN

Parameter	Form I^a^	Form IIa	Form IIb^b^	Form III^c^
Sum formula	C_20_H_21_ClO_4_	C_20_H_21_ClO_4_	C_20_H_21_ClO_4_	C_20_H_21_ClO_4_
Formula weight	360.82	360.82	360.82	360.82
Crystal system	Triclinic	Triclinic	Monoclinic	Triclinic
Space group	*P*1̄	*P*1̄	*P*2_1_/*n*	*P*1̄
*a* (Å)	8.1605 (16)	8.1328 (5)	13.619 (7)	9.4803 (6)
*b* (Å)	8.2664 (16)	8.7088 (6)	7.554 (4)	9.7605 (6)
*c* (Å)	14.511 (3)	13.6692 (9)	17.880 (9)	10.9327 (8)
*α* (°)	93.951 (3)	85.976 (6)	90	110.840 (6)
*β* (°)	105.664 (3)	84.815 (5)	92.351 (7)	90.352 (5)
*γ* (°)	96.002 (3)	74.343 (6)	90	99.701 (5)
*Z*	2	2	4	2
Cell volume	932.5 (3)	927.35 (11)	1837.91	929.53 (11)
Density	1.285	1.292	1.304	1.289 (2)
Independent reflections	4225	6241	5647	6258
*R*_int_	0.065	0.064	0.027	0.059
*R*[*I* > 2*σ*(*I*)]	0.052	0.0694	0.036	0.0653
w*R*_2_ (all)	0.147	0.1507	0.095	0.1834
Temperature (K)	193	140	100	140
Goodness of fit	1.06	1.023	1.026	1.016

a Obtained from ref. 38.

b Obtained from ref. 17.

c Obtained from ref. 11.

**Fig. 2:**
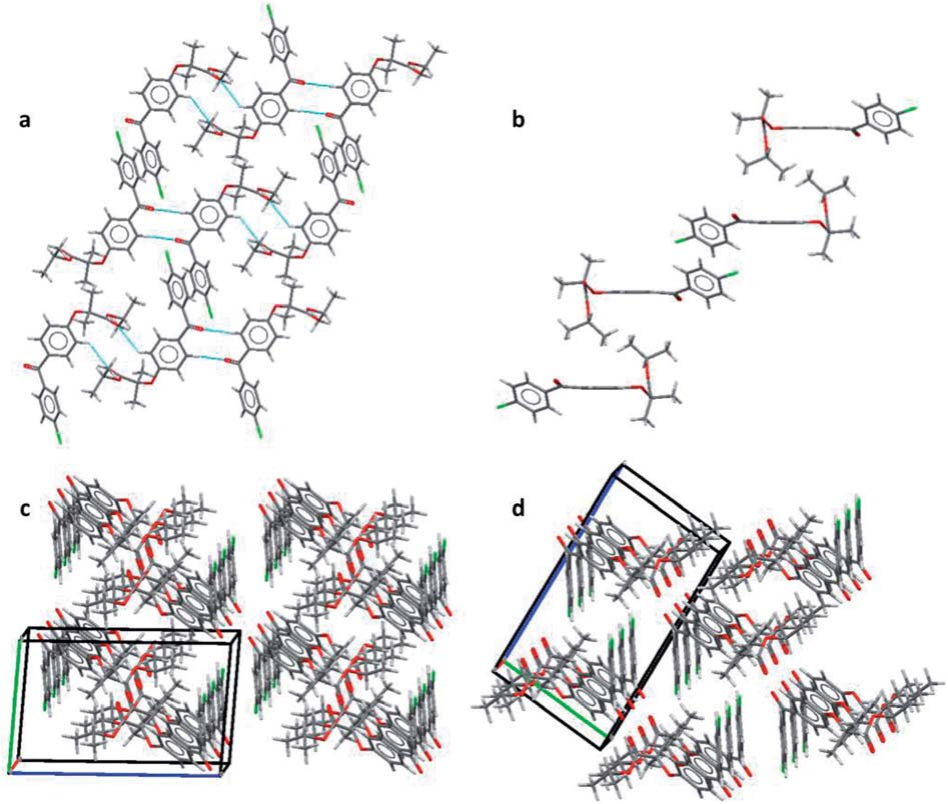
(a) Layers formed by CH⋯O interactions and π–π stacking in form IIa; (b) interactions perpendicular to the layers of form IIa; (c) packing diagram of form IIa and (d) packing diagram of form I.

Pairs of molecules in the unit cell of polymorph IIa form dimers linked by C–H⋯O hydrogen bonds between the ester carbonyl group (O3) and a hydrogen atom of the central benzene ring (H7) [d(H⋯O) = 2.662(11) Å, ∠(C–H⋯O) = 135.2(8)°]. Two of these hydrogen bonds generate a ring motif between the two molecules of the dimer. The large contact surface area between the two molecules suggests that van der Waals interactions play a significant role in stabilising the dimers (Fig. 2a).

Two principal interactions connect adjacent dimers to form layers parallel to the (111) plane: C–H⋯O hydrogen bonds and π–π interactions. The hydrogen bonds occur between an aromatic hydrogen atom in the central ring (H5) and the ketone carbonyl group of an adjacent molecule (O1) [d(H⋯O) = 2.570(17) Å, ∠(C–H⋯O) = 135.2(8)°] (Fig. 2a). Here again, two of these hydrogen bonds form a ring motif. The offset π–π interaction involves the chlorobenzene fragments of two molecules and takes place at an interplanar distance of 3.3029(8) Å between the parallel rings (Fig. 2a). The same stacking interaction was observed in form I with an interplanar distance of 3.5116(6) Å.

The most notable interaction between the layers of form IIa involves an ‘embrace’ of the alkyl groups from molecules in neighbouring layers (Fig. 2b). Interestingly, the same embrace motif is also present in form I, but there it is accompanied by a methyl to ketone C–H⋯O hydrogen bond.^11^ In form IIa the shortest H(methyl)⋯O(ketone) distance is 3.09 Å, much longer than the same contact in form I (2.56 Å). Nevertheless, the relative arrangement of the molecules remains essentially the same in both forms, suggesting that the favourable packing arrangement of the aliphatic groups is more important than the weak CH⋯O bonds. Combined, the π–π interactions and the alkyl embrace form infinite slabs of molecules (Fig. 2b), which are shared between forms I and IIa (also see Fig. 8b in (ref. 11)). The resulting similarity in the overall packing structures of both forms can be seen in Fig. 2c and d. This similarity may also explain the sensitivity of the crystallisation process to external stimuli described in the following sections.

### Crystallisation and polymorphic transformation of FEN

**Fig. 3:**
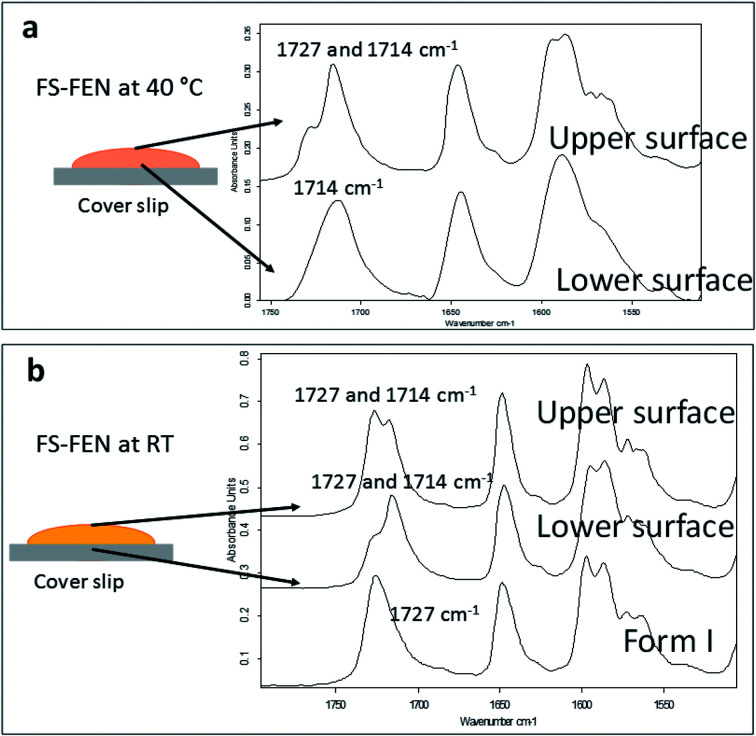
Partial ATR-FTIR spectra (1760–1500 cm^−1^) that were acquired on the upper and lower surfaces of (a) FS-FEN 40 °C and (b) FS-FEN RT. The two carbonyl stretching positions for the C_17_–O_3_ peaks at 1727 and 1714 cm^−1^ reflect a mixture between FEN forms I and IIa, whereas the carbonyl stretching of C_17_–O_3_ peak at 1714 cm^−1^ indicates FEN form IIa.

The crystallisation of FEN was first studied using the sample melted and cooled on a glass coverslip. As illustrated in Fig. 3a, the FS-FEN crystals grown at 40 °C at the open top surface (OTS) site showed a C_17_–O_3_ stretching mode at 1727 cm^−1^ which is a signature peak for form I.^16^ In contrast, the crystals grown at the interface with the glass slide showed a single C_17_–O_3_ stretching peak at 1714 cm^−1^ which is characteristic of form IIa.^16^ The FS-FEN which crystallised at room temperature showed two C_17_–O_3_ carbonyl stretching peaks (1727 and 1714 cm^−1^) at both the OTS site and the interface with the glass substrate (Fig. 3b). At the OTS site the intensity of the peak at 1727 cm^−1^ is higher than the 1714 cm^−1^ peak, indicating that the amount of form I is greater than that of form IIa. However, at the glass interface, the situation is reversed. Some care is needed in the interpretation of intensities, as there may be variations in molar absorptivity, but in the case of similar modes in the same compound a good approximation is that these will vary only slightly. These are the first set of results to indicate that the presence of OTS during the crystallisation of amorphous FEN can impact on the resulting polymorphic form of the drug. Form I preferably grows at OTS and form IIa in the bulk without OTS. This result is in good agreement with other studies, such as the surface crystallisation of indomethacin that led to the generation of a stable gamma polymorph,^23^ and it may be explained by the higher molecular mobility at the OTS, which favours the crystallisation of the thermodynamically more stable polymorph.^19–21,26^ FEN form IIa shows a preference for the site at the interface that was in contact with the glass substrate. This phenomenon is likely due to the substrate induced phase (SIP) effect. The SIP effect has been reported to influence the polymorphic form of APIs.^23^ Reischl *et al.* reported the use of SIP to induce a metastable polymorph of phenytoin. The authors explained that the surface of the substrate has a large effect on the phenytoin crystal alignment that leads to a formation of the metastable polymorph of phenytoin.^25^ Therefore, it is possible to speculate that the origin of FEN form IIa is from the SIP effect of the glass substrate that induces the FEN molecular alignment into the polymorph IIa.

**Fig. 4:**
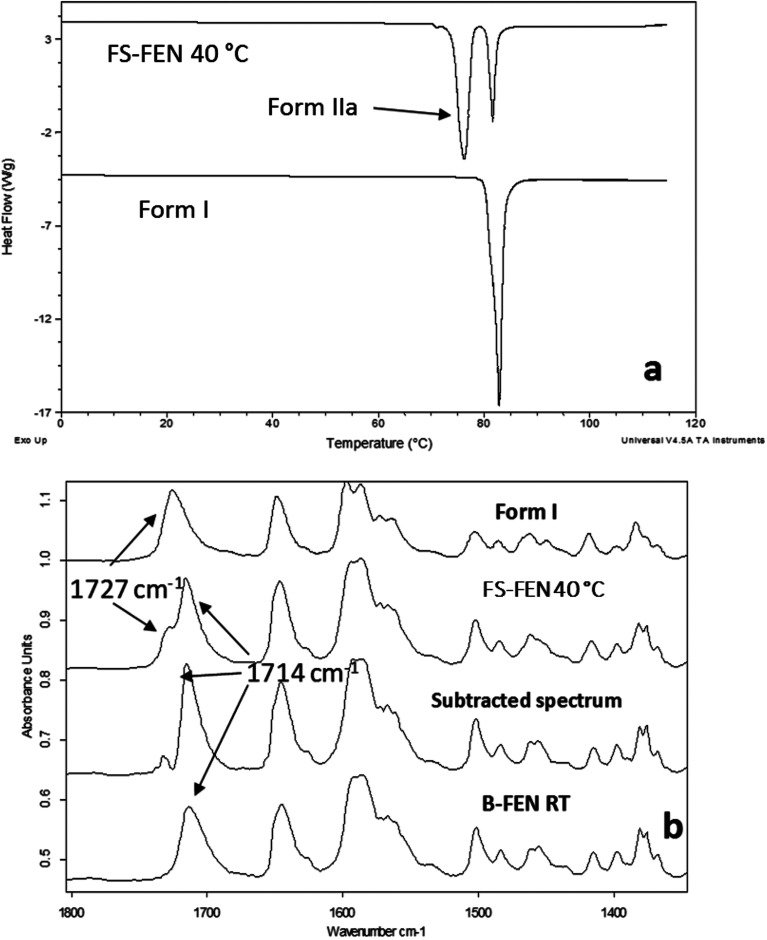
(a) Melting points of FS-FEN 40 °C in comparison to the reference form I; (b) partial ATR-FTIR spectra of: reference form I; FS-FEN 40 °C; FS-FEN 40 °C after subtracting 40% of the intensity of the form I spectrum; B-FEN crystallised at room temperature.

As shown in Fig. 4a, the amorphous FEN, which crystallised at 40 °C with an OTS (FS-FEN 40 °C), had three endothermic melting peaks for FEN at 70, 74 and 80 °C. The peak at 70 °C has an extremely low enthalpy value indicating a trace quantity of this form, which could be a trace of form IIb. However, as the melting point of form IIb was not reported in the literature, this could not be confirmed. The two main melting peaks are the FEN forms IIa and I.^14,16^ The literature does not report the melting enthalpy of the pure FEN form IIa. However, as discussed in the previous section, the single crystal structures of forms I and IIa are similar. It was thus expected that FEN forms I and IIa would demonstrate similar melting enthalpies. This allows the rough estimation of the amount of each form from the melting enthalpy values. The melting enthalpy of form IIa peak is 57.9 ± 1.8 J g^−1^ whereas form I had a melting enthalpy of 27.9 ± 5.6 J g^−1^, indicating that form IIa is the dominant polymorphic form under these conditions.

In the B-FEN samples, a different crystallisation behaviour was observed. The ATR-FTIR spectrum of the B-FEN which crystallised between coverslips at room temperature (B-FEN RT) was examined immediately after removing the cover slips with careful avoidance of any other physical contact with the samples. The spectrum is identical with the spectrum of form IIa (Fig. 4b). Therefore, it can be concluded that covering the sample (B-FEN RT) resulted in a significantly increased amount of form IIa crystallisation in comparison to FS-FEN. DSC was not performed on these samples, as the preparation of the sample for DSC requires removal by scratching the crystals off the coverslip and this process is known to induce the conversion of form IIa to form I (Fig. S3†).

### Effect of crystal growth temperatures on FS-FEN crystallisation

**Fig. 5:**
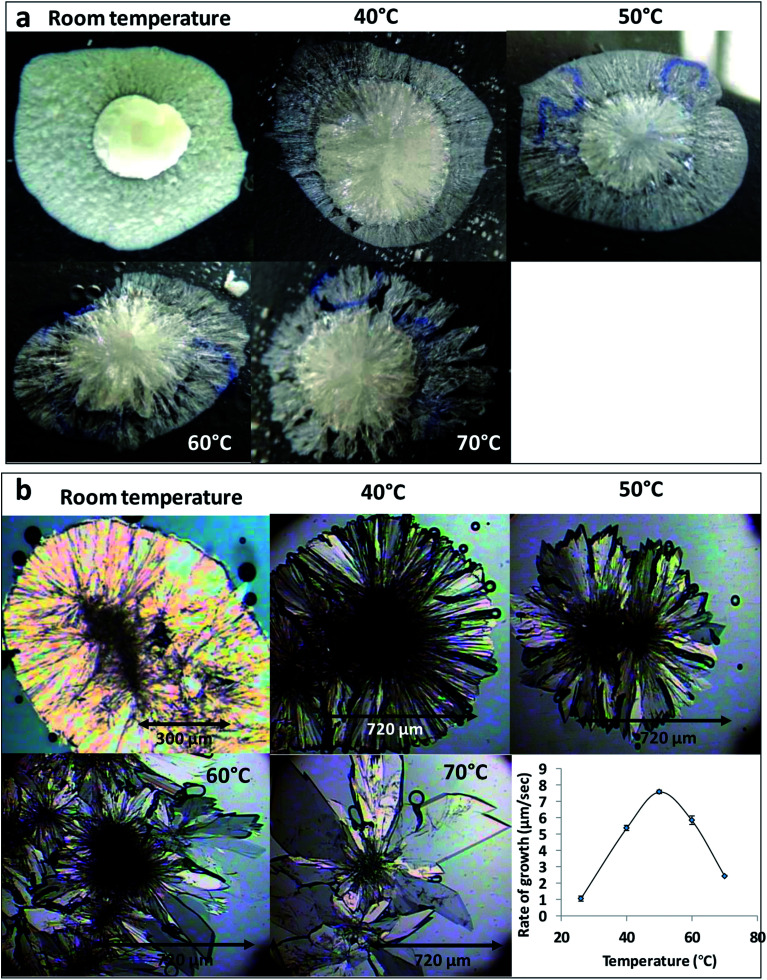
(a) Optical images of FS-FEN growth at different temperatures and (b) PLM images of B-FEN growth at different temperatures and the change in crystal growth rate associated with the temperature effect.

**Fig. 6:**
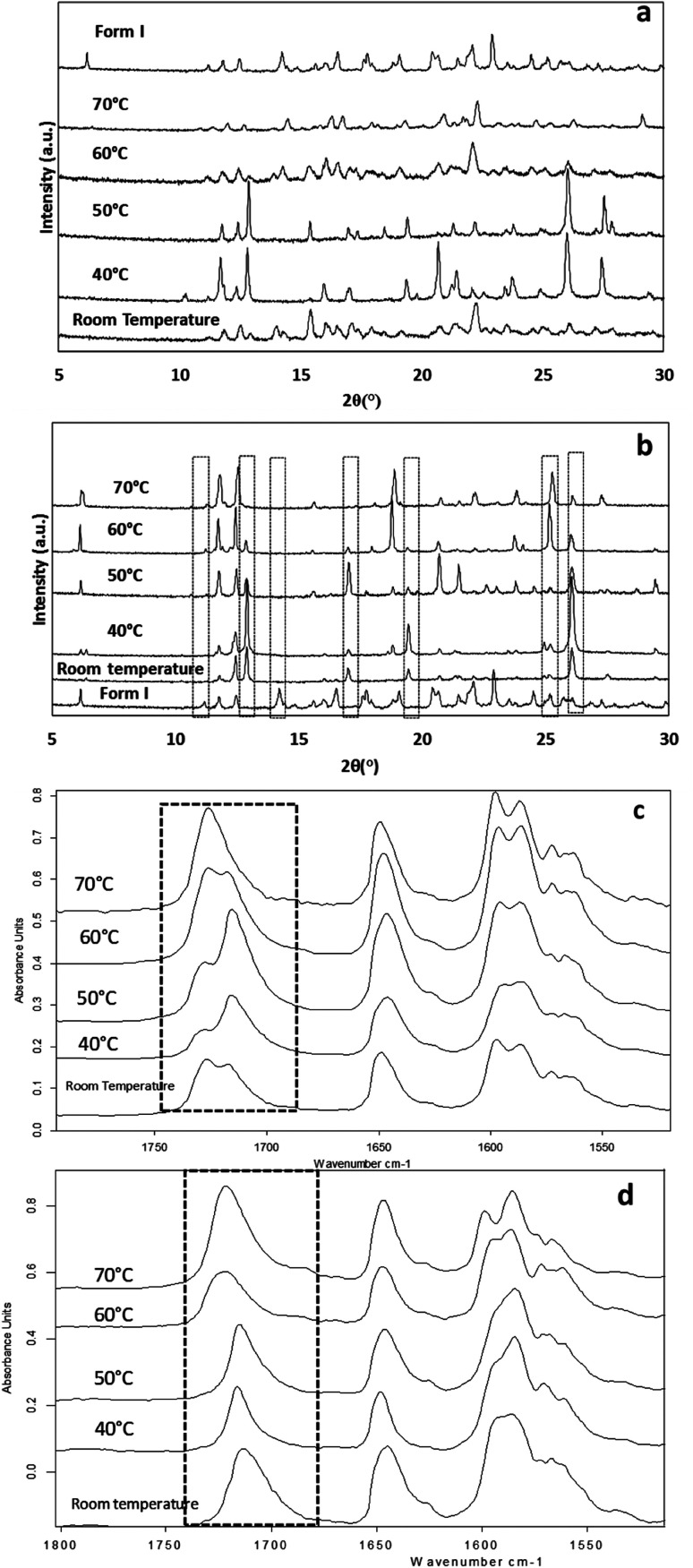
PXRD patterns of (a) FS-FEN and (b) B-FEN at different crystal growth temperatures and partial ATR-FITR spectra (1800–1500 cm^−1^) of (c) FS-FEN and (d) B-FEN that were crystallised at various growth temperatures (70, 60, 50, 40 °C and room temperature).

The effect of temperature on the crystal growth of FEN was first indicated by the difference between the FS-FEN 40 °C and FS-FEN RT, as discussed in the previous section. Therefore, the effect of temperature on the crystal growth at OTS was studied by storage at room temperature (22 °C), 40, 50, 60 and 70 °C for 30 minutes immediately after the crystallisation was initiated by scratching. The crystallisation progressed rapidly as reported by Amstad^12^ and within 30 minutes all amorphous samples appeared to be fully crystallised. At room temperature, the continuous growth of fine opaque crystals can be seen underneath the white crystal cluster in the centre where the crystallisation was initiated by mechanical scratching. However, crystal growth at the temperatures of 40, 50 and 60 °C led to the growth of a layer of transparent spherulite crystals beneath the opaque central crystal cluster that was formed first (Fig. 5a). The PXRD patterns of the FS-FEN RT and FS-FEN 60 °C (Fig. 6a) contain peaks from form I and form IIa peaks that match the ones reported by Di Martino and co-workers. This further confirms that the crystals were a mixture of forms I and IIa.^16^ The PXRD patterns of the FS-FEN crystallised at 40 and 50 °C are clearly not pure FEN form I, and most diffraction peaks are at similar positions as those of B-FEN RT, including the diffraction peaks at 12.9, 17.6, 19.5, 26.0 and 27.6° (Fig. 6b). As B-FEN RT was confirmed being mostly form IIa by ATR-FTIR, this result indicates that more form IIa than form I crystallised at 40 and 50 °C. This is further confirmed by DSC and ATR-FTIR results.

**Fig. 7:**
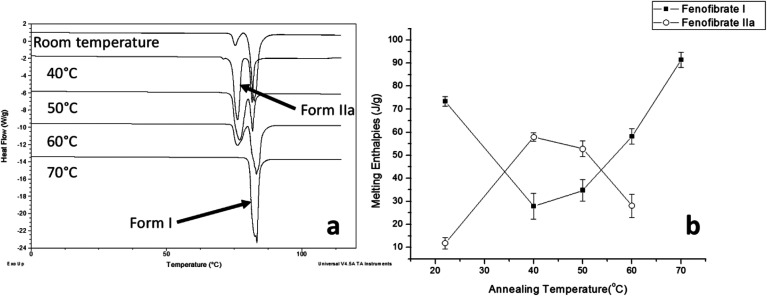
(a) DSC thermogram demonstrating melting enthalpies of FS-FEN, which were incubated at different temperatures during their crystallisation; (b) changes in the melting enthalpies of FEN forms I and IIa with incubating temperature.

The DSC detected two main melting transitions at 74 and 80 °C in the FS-FEN samples that crystallised at room temperature, 40, 50 and 60 °C, which indicates the co-existence of FEN forms IIa and I and is consistent with the PXRD results (Fig. 7a). The trace amount of possible form IIb with the melting at 70 °C observed in the FS-FEN at 40 °C was not identified in the PXRD diffraction pattern due to the low quantity of this material. From the melting enthalpy values, one can obtain a semi-quantitative comparison of the amount of form I and form IIa obtained in the samples that were grown at different temperatures (Fig. 7b). At 40 and 50 °C, more form IIa was produced than form I. For the samples grown at room temperature and 60 °C, form I was the dominant polymorph. The ATR-FTIR findings agree with the DSC results. The two ester carbonyl stretching C_17_–O_3_ peaks at 1714 and 1727 cm^−1^ were observed in the ATR-FTIR spectra of the samples grown at room temperature, 40, 50 and 60 °C, which confirmed the co-existence of form I and form IIa (Fig. 6c). The ester carbonyl peak at 1714 cm^−1^ was more intense than that at 1727 cm^−1^, confirming that at 40 and 50 °C form IIa was the dominant form. In contrast the FS-FEN grown at room temperature and 60 °C showed a greater intensity of the ester carbonyl peak at 1727 cm^−1^ indicating the domination of the crystallisation of FEN form I under these conditions.

As the growth temperature was increased to 70 °C, the crystal habits of both the top and bottom surfaces of the growth gradually changed to blade-like shapes, as seen in Fig. 5a. At 70 °C, only form I with a single melting point at 80 °C was detected by DSC. This was further confirmed by the presence of the single form I ester carbonyl stretching at 1727 cm^−1^ in the ATR-FTIR spectrum and a form I PXRD diffraction pattern (Fig. 6a).

### Effect of crystal growth temperatures on B-FEN crystallisation

For the B-FEN samples, the crystal morphology changed significantly with changing the growth temperature. At room temperature, the crystals spread rapidly in a symmetrical spherulitic manner during growth. When 40, 50, 60 and 70 °C were used as the crystal growth temperatures, the FEN crystals became a blade-like shape and expanded asymmetrically (Fig. 5b). The crystal growth rate also showed high sensitivity to the changes in the incubation temperature. As seen in Fig. 5b, the crystal growth rates were 1.05, 5.37, 7.58, 5.83 and 2.4 μm s^−1^ at room temperature (22 °C), 40, 50, 60 and 70 °C, respectively. The maximum crystal growth rate of the FEN was observed at 50 °C. This agrees with Amstad's study that the maximum crystal growth rate of FEN is at 50 °C.^12^

**Fig. 8:**
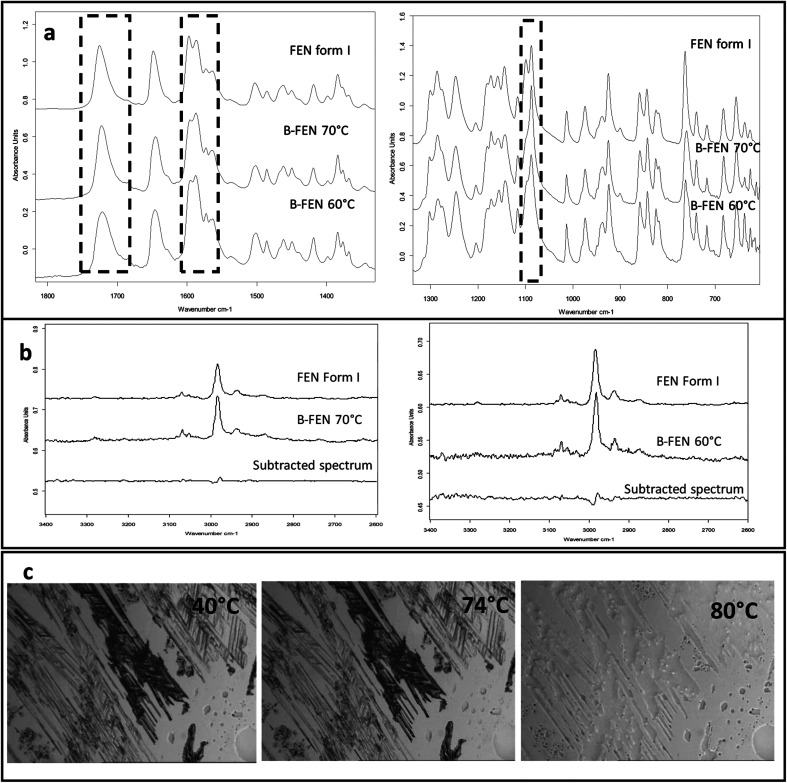
ATR-FTIR spectra of (a) B-FEN 60 and 70 °C in comparison to the reference form I spectrum (1800–600 cm^−1^); (b) the subtracted spectra of B-FEN 60 and 70 °C from the form I spectrum at 3400 to 2600 cm^−1^; (c) HS-PLM images of B-FEN 70 °C heated to 40, 74 and 80 °C.

ATR-FTIR results confirm that the B-FEN crystallisation at 40 and 50 °C resulted in predominately FEN form IIa (Fig. 6d). The ATR-FTIR spectra of B-FEN 60 and 70 °C closely resembled the spectrum of FEN form I, but with differences in the ester carbonyl peak which was shifted to 1721 cm^−1^. There were also slight changes in the intensity of the C=C stretch of the benzene ring at 1520 and 1463 cm^−1^ (Fig. 8a) and the benzene ring in-plane deformation vibration peak at 1098 cm^−1^ was absent (Fig. 8b). The spectrum subtraction of the B-FEN 60 and 70 °C from FEN form I was performed by ensuring that the resultant subtracted spectrum had a flat base line in the methyl stretching region (3400–2600 cm^−1^). This was chosen as the spectra of forms I and IIa do not differ in this region. The subtraction resulted in an intensity close to zero. The small intensities observed are attributed to orientation effects with respect to the refracting ATR crystal resulting in a small apparent peak shift.^32^

The PXRD results also show the changes in the diffraction patterns of B-FEN with changes in the growth temperatures (Fig. 6b). Example diffraction peaks are highlighted in Fig. 6b and they show the clear differences between the known form I and the growth of the B-FEN at different temperatures. The diffraction pattern of B-FEN grown at 40 °C indicates the crystallisation of mostly FEN form IIa with a small quantity of form I. As the ATR-FTIR data shows no indication of form I, it is likely that the small amount of form I was converted from form IIa during the PXRD sample preparation, during which an OTS had to be introduced. The PXRD data of B-FEN grown at 70 °C PXRD is consistent with FEN form I. In both cases, there are few peaks missing in comparison to the literature data. This is attributed to the orientation effect, which can be seen in both cases with (001) plane as preferred orientation for both forms. PXRD may therefore, not be the most suitable technique to examine the B-FEN samples. Hot-stage microscopy was performed to clarify the polymorph of the B-FEN 70 °C by determining its melting point. The result demonstrated that the B-FEN 70 °C melts at 80 °C, which is the same as the form I melting point and confirms it being FEN form I (Fig. 8c).

### Physical stability of FEN form IIa formed with and without OTS

**Fig. 9:**
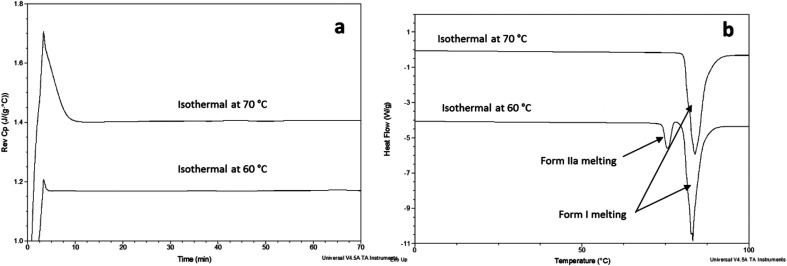
(a) Isothermal MTDSC thermogram of FS-FEN IIa rich crystals at 60 and 70 °C for 120 min and (b) DSC heating of a FS-FEN 40 °C sample after isothermal MTDSC at 60 and 70 °C.

**Fig. 10:**
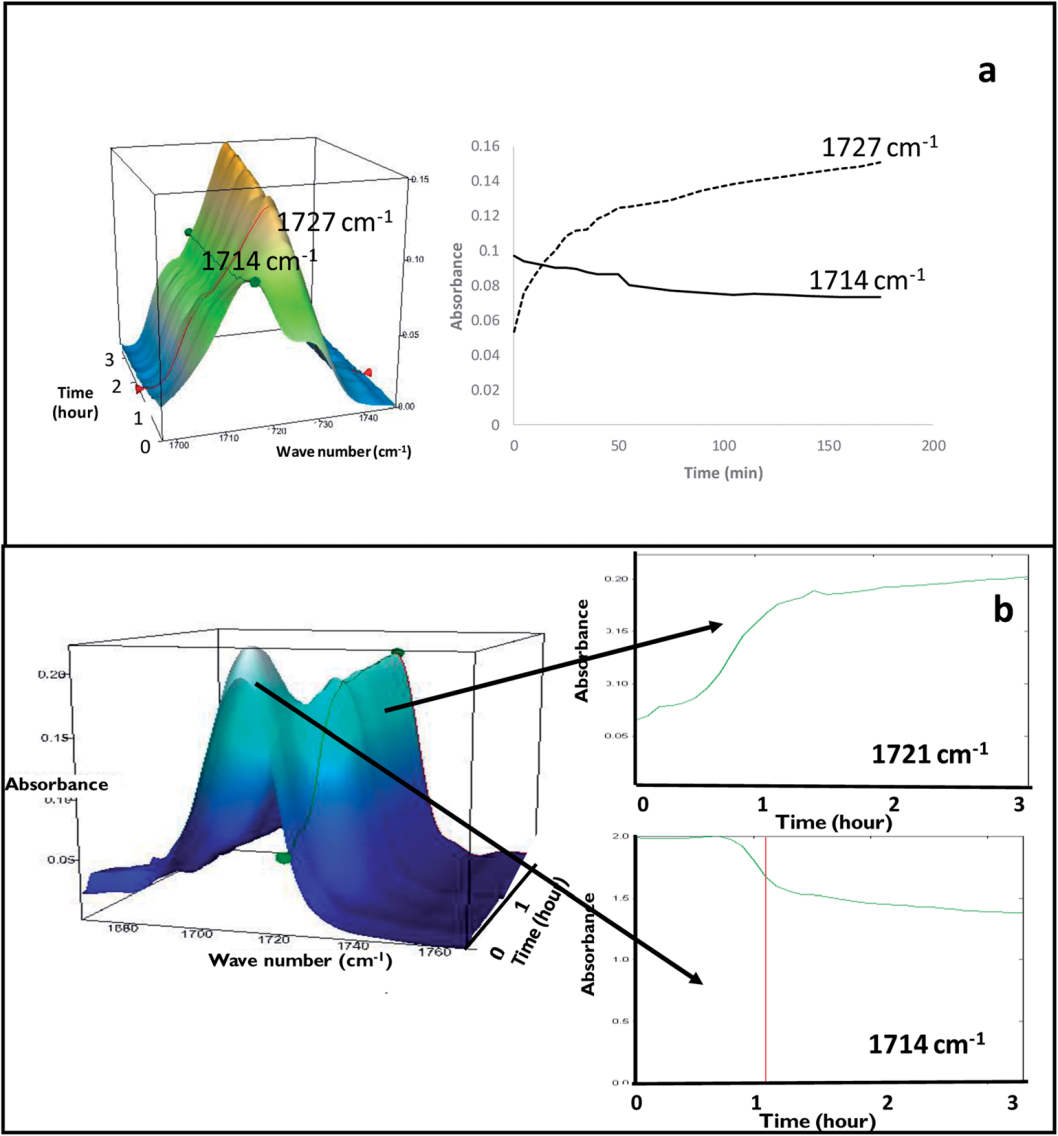
(a) Peak intensity changes of ester carbonyl stretching peak at 1714 cm^−1^ (form IIa) shifted to 1727 cm^−1^ (form I) during 3 hours annealing at 70 °C of FS-FEN 40 °C indicating the transformation of form IIa to form I (b) peak intensity changes in ATR-FTIR spectra of B-FEN after removing coverslips and placing on the variable temperature ATR-FTIR for 3 hours annealing at 70 °C. It should be noted that the characteristic peak of FEN form II at 1714 cm^−1^ gradually shifted to 1721 cm^−1^ indicating the polymorphic conversion of form IIa to I.

FS-FEN 40 °C was chosen as the representative sample to study the physical stability of FEN form IIa because it contains the greatest amount of the form IIa. The stability was monitored during six months at room temperature using PXRD. There is no clear evidence of the continuous polymorphic transformation of form IIa to I in the mixtures at room temperature (Fig. S1†). However, the conversion to form I can be accelerated by applying thermal annealing. Isothermal MTDSC was used to further probe the effect of annealing temperature on the conversion of form IIa to form I. It has been reported previously that a change in the *C*_p_ during an isothermal DSC experiment is related to polymorphic conversion.^33^ As seen in Fig. 9a, a more significant surge of the reversing *C*_p_ signal was observed at the beginning of the annealing period at 70 °C than at 60 °C (completed within 11.3 ± 1.2 minutes for annealing at 70 °C and 4.8 ± 0.2 minutes at 60 °C). The peak observed in the reversing *C*_p_ signal is associated with the polymorphic conversion of form IIa to form I. A standard DSC was used to confirm the nature of the *C*_p_ change after the isothermal MTDSC experiment. Only the melting of form I was detected in the FS-FEN 40 °C samples that had been annealed at 70 °C (Fig. 9b). This indicates that the change in the *C*_p_ is associated with the complete polymorphic transformation of form IIa to form I; whereas, the melting of form IIa can still be observed in the sample that annealed at 60 °C, but with a reduced enthalpy value and accompanied by the melting of form I. The reduced enthalpy of FEN form IIa is again associated with an incomplete conversion of form IIa to form I during annealing at 60 °C (Fig. 9b). Variable temperature ATR-FTIR spectroscopy of the FS-FEN 40 °C sample that annealed at 70 °C revealed the transformation of the two ester carbonyl stretching peaks at 1714 and 1727 cm^−1^ to a single peak at 1727 cm^−1^ which agrees well with the DSC results confirming the conversion of form IIa to form I (Fig. 10a). A similar transformation occurs when B-FEN is annealed at 70 °C (Fig. 10b).

For B-FEN samples, after the complete crystallisation at room temperature, PXRD was used to monitor the six-month physical stability of form IIa stored at room temperature. The form IIa is stable for at least six months at room temperature after the top substrate is removed without conversion into form I (Fig. S2†). This indicates that at room temperature, form IIa is relatively stable, which is confirmed by the single crystal structural study.

### ‘Switching’ of polymorphic form growth

**Fig. 11:**
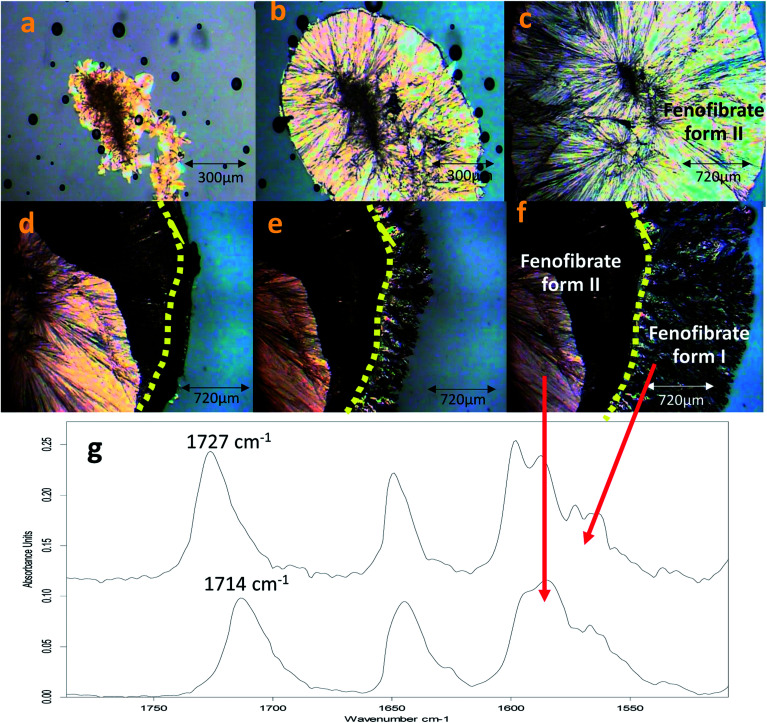
Images of FEN crystal growth mode being switched from form IIa to form I after top coverslip removal. (a–c) FEN form IIa crystals grown between sandwiched coverslips at room temperature over 17 minutes (*a* = initial, *b* = 5 min, *c* = 15 min); (d–f) following top cover slip removal, form IIa crystal growth was terminated and form I crystallisation occurred throughout the remaining amorphous FEN (*d* = initial, *e* = 5 min, *f* = 15 min); and (g) spherulite and needle-like crystalline regions were distinguished as two separate domains using ATR-FTIR in which the ester carbonyl stretching peaks at 1714 and 1727 cm^−1^ indicates the presence of forms IIa and I, respectively.

The conversion of form IIa to form I can be induced by external mechanical stress, such as scratching with a stainless-steel spatula, or thermal treatment (Fig. S3†). In addition to this, the significance of OTS on the crystallisation of FEN polymorphic forms from the melt is further demonstrated by a ‘switching’ effect of the polymorphic growth. It was observed that if there was still non-crystallised amorphous material available in B-FEN samples, removing the cover slip led to the termination of the crystallisation to form IIa, and the growth of form I. As seen in Fig. 11a–c, the continuous growth of FEN form IIa from the melt was observed between coverslips. The spherulite form IIa crystals, which were identified by the ester carbonyl vibration at 1714 cm^−1^ using ATR-FTIR spectroscopy, expand radially from the nucleation site (initiated by surface scratching) throughout the melt. The removal of the top coverslip before the completion of the crystallisation terminated the expansion of the form IIa (Fig. 11d). Instead, needle-like clusters of crystals could be seen growing rapidly on the edge of the form IIa crystals (Fig. 11e and f). These needle-like crystals were identified as form I by the shift of the ester carbonyl stretching peak to 1727 cm^−1^ using ATR-FTIR spectroscopy (Fig. 11g). This phenomenon, in which the crystalline form IIa initiates form I, is likely to be due to cross-nucleation.^26,34–37^ This is a phenomenon in which one crystalline polymorph is nucleated by another polymorph. Tao *et al.* observed that the seed crystals of mannitol forms β and δ induced the crystallisation of mannitol form α at a temperature below 150 °C. While mannitol form β was the only form that crystallised at a temperature greater than 150 °C.^26^ Gunn *et al.* reported the crystallisation and growth of nifedipine metastable β and X forms at growth temperatures below 110 °C, whereas pure stable α form of nifedipine was obtained at growth temperatures above 120 °C.^35^ Hence, the cross-nucleation of mannitol and nifedipine is temperature dependent.^35,36^ To the best of our knowledge, this ‘switching’ effect of the crystal forms provided by the presence of OTS has not been reported previously.

## Conclusion

This study demonstrated that the availability of an OTS during crystallisation is a factor that strongly impacts the polymorphic form selection of the super cooled liquid of FEN. Moreover, the use of a thermal treatment in addition to an OTS can fine tune the selection of the crystallisation of FEN into form I and form IIa. The crystal growth mode switching form IIa to form I occurs when an OTS is introduced to the incomplete crystallisation of form IIa. This indicates that the crystallisation of form I is highly dependent on the OTS. This study reports for the first time the crystallographic data of metastable FEN form IIa, which confirmed the similarity of it to the molecular packing of the stable form I and clarified the confusion between the FEN form IIs in the literature. This study has produced new insights into how to use an OTS to manipulate and control the crystallisation of FEN which will allow improved polymorphic control during crystallisation of FEN.

## Conflicts of interest

There are no conflicts to declare.

## Supplementary Material

RA-008-C8RA01182F-s001

RA-008-C8RA01182F-s002
