# Supplementary material for: Towards controlling the crystallisation behaviour of fenofibrate melt: triggers of crystallisation and polymorphic transformation
Source: RSC Adv. 2018 Apr 10;8(24):13513–25. doi: 10.1039/c8ra01182f (PMC9079832; doi:10.1039/c8ra01182f)
Supplement: RA-008-C8RA01182F-s001 [file RA-008-C8RA01182F-s001.pdf]

Electronic Supplementary Material (ESI) for RSC Advances.
This journal is © The Royal Society of Chemistry 2018

Supplementary Materials

Towards Controlling the Crystallisation Behaviour of Fenofibrate Melt: Triggers of crystallisation and polymorphic transformation

Pratchaya Tipduangta[1,3], Khaled Takieddin[1], László Fábián[1], Peter Belton[2] and Sheng Qi[1]*

School of Pharmacy University of East Anglia, Norwich, Norfolk, UK, NR4 7TJ [1]

School of Chemistry University of East Anglia, Norwich, Norfolk, UK, NR4 7TJ [2]

Faculty of Pharmacy Chiang Mai University, Chiang Mai, Thailand 50200[3]

Email*: Sheng Qi sheng.qi@uea.ac.uk

List of content:

Figure S1. PXRD diffraction patterns of the OTS-FEN samples crystallised at 40 °C and stored under ambient conditions: fresh (A), one month (B), three months (C), six months (D) and intact crystalline form I (E).

Figure S2. PXRD diffraction patterns of the B-FEN samples stored under ambient conditions: fresh (A), one month (B), three months (C) and six months (D).

Figure S3. DSC result of the B-FEN sample that was scratched to induce the crystallisation**.**

Figure S1 shows the DSC profile of B-FEN. It can be clearly seen that the form IIa melted at approximately 74° C which immediately recrystallized into form I and melted with the rest of form I. Judging by the significantly higher enthalpy value of the form I melting peak, it is certain that the original sample after scratching contained significant amount of form I before the DSC heating.

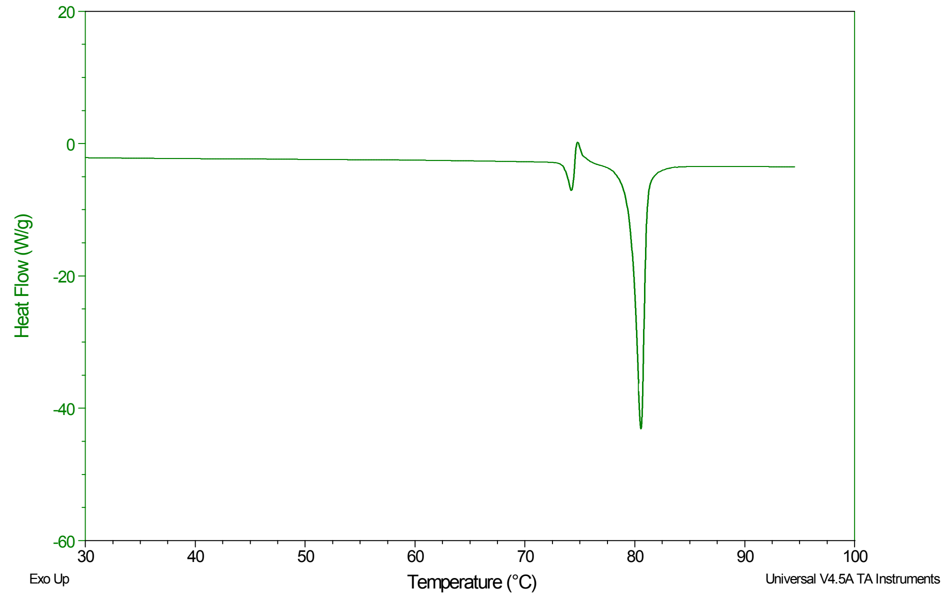

Figure S1 DSC result of the B-FEN sample that was scratched to induce the crystallisation.

Figure S2 showed little changes in the diffraction pattern of the OTS-FEN samples crystallised at 40 °C within the 6 storage at room temperature. After 6-month storage, it still contains significant amount of form IIa. Little changes are observed in the peak intensities indicating no continuous polymorphic transformation of form IIa to I in the mixtures at room temperature.

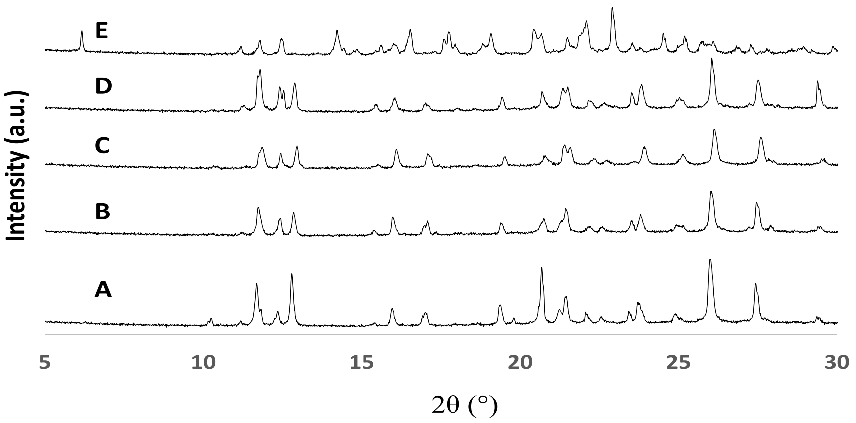

Figure S2 PXRD diffraction patterns of the OTS-FEN samples crystallised at 40 °C and stored under ambient conditions: fresh (A), one month (B), three months (C), six months (D) and intact crystalline form I (E).

For B-FEN samples, after the complete crystallisation at room temperature, PXRD was used to monitor the 6-month physical stability of form IIa stored at room temperature. Figure S2 shows no significant changes in the PXRD diffraction pattern indicating the form IIa is stable for at least six months at room temperature after the top substrate is removed without conversion into form I.

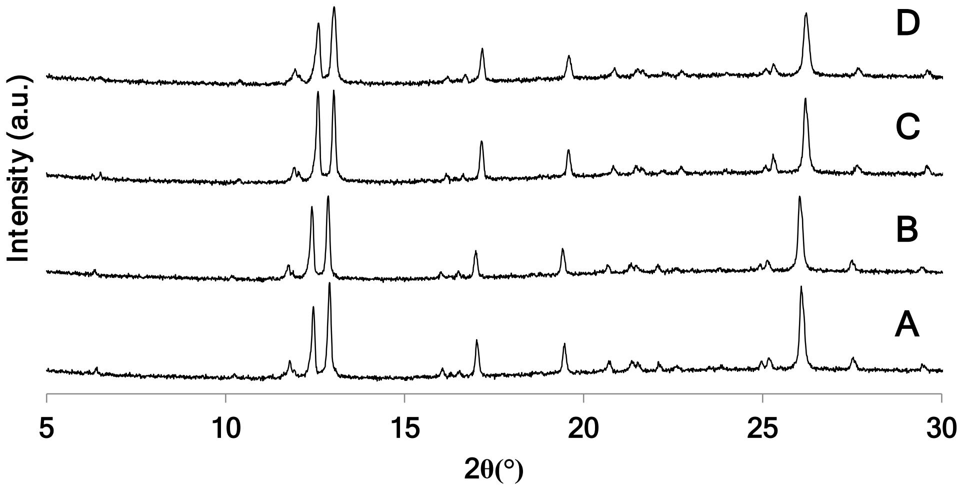

Figure S3 PXRD diffraction patterns of the B-FEN samples stored under ambient conditions: fresh (A), one month (B), three months (C) and six months (D).
